# Through-Ice Acoustic Communication for Ocean Worlds Exploration

**DOI:** 10.3390/s24092776

**Published:** 2024-04-26

**Authors:** Hyeong Jae Lee, Yoseph Bar-Cohen, Mircea Badescu, Stewart Sherrit, Benjamin Hockman, Scott Bryant, Samuel M. Howell, Elodie Lesage, Miles Smith

**Affiliations:** NASA/Jet Propulsion Laboratory, California Institute of Technology, Pasadena, CA 91109, USA; yoseph.bar-cohen@jpl.nasa.gov (Y.B.-C.); mircea.badescu@jpl.nasa.gov (M.B.); stewart.sherrit@jpl.nasa.gov (S.S.); benjamin.j.hockman@jpl.nasa.gov (B.H.); scott.h.bryant@jpl.nasa.gov (S.B.); samuel.m.howell@jpl.nasa.gov (S.M.H.); elodie.lesage@jpl.nasa.gov (E.L.); miles.smith@jpl.nasa.gov (M.S.)

**Keywords:** acoustics, communication, Ocean Worlds missions, planetary exploration, Europa

## Abstract

Subsurface exploration of ice-covered planets and moons presents communications challenges because of the need to communicate through kilometers of ice. The objective of this task is to develop the capability to wirelessly communicate through kilometers of ice and thus complement the potentially failure-prone tethers deployed behind an ice-penetrating probe on Ocean Worlds. In this paper, the preliminary work on the development of wireless deep-ice communication is presented and discussed. The communication test and acoustic attenuation measurements in ice have been made by embedding acoustic transceivers in glacial ice at the Matanuska Glacier, Anchorage, Alaska. Field test results show that acoustic communication is viable through ice, demonstrating the transmission of data and image files in the 13–18 kHz band over 100 m. The results suggest that communication over many kilometers of ice thickness could be feasible by employing reduced transmitting frequencies around 1 kHz, though future work is needed to better constrain the likely acoustic attenuation properties through a refrozen borehole.

## 1. Introduction

The advancement of technology for deep subsurface communication through tens of kilometers of ice has significant potential in supporting the investigation of subsurface liquid water on Ocean Worlds, including Europa and Enceladus. Direct access to subsurface liquid water would address a fundamental question of the National Aeronautics and Space Administration’s (NASA) planetary exploration program with regards to whether life has ever arisen elsewhere in our solar system. Europa’s deep subsurface and internal ocean are among the most promising places to search for life in the Solar System [1,2,3]. NASA’s Jet Propulsion Laboratory (JPL) is currently developing robotic systems designed to explore Ocean Worlds, and “Cryobot” melt probes are the leading mission architectures—robots designed to melt through the ice using the waste heat from radioisotope power systems [4,5,6].

The communication system between the lander and the Cryobot through the ice shell must address the unique challenges in each of the crust layers in Europa. The total thickness of Europa’s ice shell is fundamentally uncertain, though many studies have used various modeling techniques to constrain the likely range from a few kilometers to a few tens of kilometers. A Bayesian heat flow analysis suggests a maximum likelihood of about 24 km [7]. The ice shell is also widely believed to consist of two primary layers: a cold and brittle region overlaying a warm, ductile region that may be nearly isothermal (at about 250 K), both of which may range in thickness from about 5 to 40 km. Each region poses unique challenges for communication between the Cryobot and the surface, particularly at the top, a cold and brittle region where tidal deformations could induce low-magnitude shear stresses (around 100 kPa), leading to crack formation. Consequently, this makes a solely tethered communications system too risky for the success of the Cryobot mission [8,9,10,11].

One of the proposed solutions currently in development is a wireless communication link through the ice that consists of a series of relay transceiver pucks to complement a deployed fiber optic tether. These transducers use radio frequency (RF) communications; however, RF signals become highly attenuated in warm, salty, or wet ice conditions that exist in the lower ductile region. One solution is to use acoustic communication within the warm ice zone and briny ice regions where RF is highly attenuated; however, the current challenge lies in the lack of an acoustic communication system capable of effectively transmitting signals through ice. This led to the need for the development of specialized ice-penetrating acoustic transducers to address this technological gap.

In this paper, the progress made in advancing acoustic communication through ice environments is presented. A description of the acoustic communication system used for this study is presented, followed by a discussion of laboratory testing results. Due to the limitations of the laboratory test setup, which spans only a few meters of ice, field validation becomes essential. Thus, the breadboard transceivers were field-tested at Matanuska Glacier in Alaska to assess the viability of acoustic communication through ice. The field site was selected for its ability to have the test in ice over 100 m, as well as its accessibility. The results of the field test and lessons learned for further advancement of the technology are discussed in this paper.

## 2. Design and Construction

The breadboard configuration for this task is based on the mission formulation study conducted at JPL, in which a notional Cryobot system architecture was designed to penetrate Europa’s ice shell. The communication elements in the Cryobot architecture are an RF patch antenna operating at approximately 100 MHz–1 GHz (communicating with the surface) and three large ring-type piezoelectric transducers capable of achieving frequencies as low as 1 kHz while still being small enough to be packed into the volume-constrained aft section of the Cryobot [6].

The design goal considers the operating frequency of the acoustic transducer as well as the size of electronic components to enable effective proof-of-concept communication through many kilometers of ice. Several underwater piezoelectric transducer types, such as piston, ring, flexural disks, and flextensional, were considered as candidates for the design of the transceivers. The piston-type transducer offers high directionality and a high source level compared to other transducer designs; however, the operating frequency of this type of transducer is limited, generally higher than 10 kHz. Flextensional-type transducers with a metallic flexure can generate low-frequency (less than 1 kHz) waves; however, their performance in high hydrostatic pressure conditions remains uncertain and needs future study. The ring-type free-flooded transducer was selected because of its capability to generate low frequencies (1~10 kHz) in compact form. Most importantly, free-flooded rings have a high hydrostatic pressure tolerance that permits their use at Europa-equivalent depths (~40 km) that other transducer types cannot withstand.

**Figure 1:**
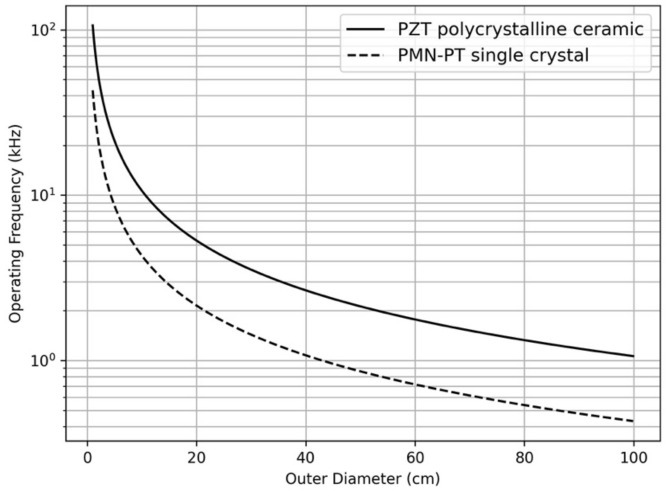
The relationship between the outer diameter of the ring transducer and the operating frequency depending on the type of piezoelectric material.

Figure 1 shows the relationship between the operating frequency (resonance frequency) of a ring-type transducer and its diameter depending on the piezoelectric material type, where the ring mechanical resonance (*f*) occurs when the wavelength is equal to the circumference of the ring (i.e., f.D = c/π, where c is wave speed) [12]. The results suggest that to achieve a 1~2 kHz ring transducer, conventional lead zirconate titanate (PZT) material requires a diameter exceeding 100 cm, whereas lead magnesium niobate–lead titanate (PMN-PT)-based single crystals can reduce half the size due to their relatively lower elastic constants, resulting in a lower wave speed in PMN-PT. Initial investigations suggest that achieving a radiated acoustic power of 10 W at a 1 kHz operating frequency enables acoustic communication over distances exceeding 10 km based on recent attenuation data in the South Pole [13]. A transducer with an outer diameter of 22 cm and a height of 20 cm demonstrates capability for meeting these specifications; however, in this study, adjustments were made to the size and frequency of the acoustic transducers to accommodate cost and size constraints for laboratory testing.

**Figure 2:**
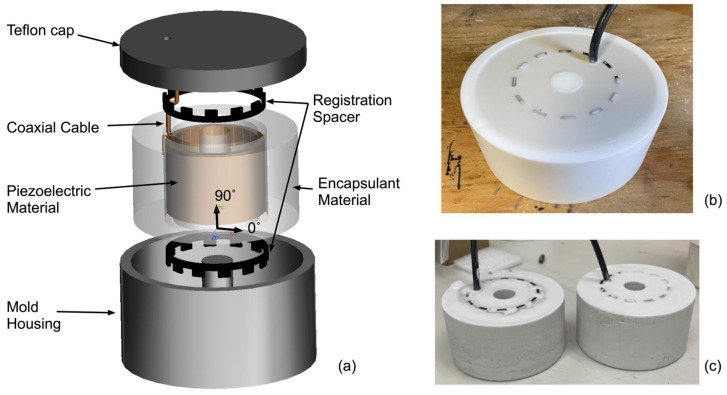
(**a**) Exploded view of the acoustic transducer design; (**b**) image of the acoustic transducer with a mold housing; and (**c**) image of the fabricated acoustic transducer after the removal of the mold housing.

For the development of an acoustic transducer, free-flooded ring transducers were designed, specifically tailored for operating in ice environments, using a mixture of cryogenic epoxy materials and alumina for encapsulant materials. This results in improved acoustic impedance matching with ice. Note that the commercially available free-flood ring transducer is specifically designed and optimized for use in water media, making it unsuitable for applications in ice environments. Specifically, the typical housing material (e.g., Delrin, neoprene rubber) and encapsulant material (e.g., silicon oil or polyurethane), which are effective in water, are not optimal for use in ice environments. The acoustic transducer design and photos of fabricated acoustic transducers are shown in Figure 2.

**Figure 3:**
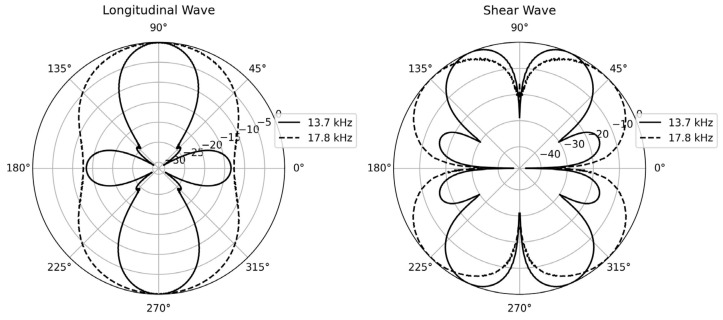
Simulated directivity patterns of longitudinal and shear waves generated by the acoustic transducer within ice using FEM.

The directional characteristics of the transducers in ice media have been investigated using FEM. For a ring transducer, both longitudinal and shear waves are generated as a result of cavity resonance. This phenomenon occurs when the ring extends, the surrounding media is compressed, and the media inside expands, and vice versa. The combination of motions of the inner and outer media causes resonance mode, leading to both longitudinal and shear wave generation along the axial axis. Figure 3 shows the results, exhibiting radiation patterns of bulk longitudinal and shear acoustic waves generated by the transducer at 13.7 kHz and 17.8 kHz, respectively. The result indicates that the bulk longitudinal wave primarily travels along the axial direction of the ring (90°), while shear waves do not propagate in the axial direction; instead, they propagate at a 45-degree angle. This beam pattern is advantageous for communication purposes in open spaces as the main longitudinal wave experiences less interference from the shear wave.

## 3. Drive Electronics

The main goal of the driving electronics development is to achieve programmable control of the frequency, baud rate, and modulation schemes while having a small, cost-effective, and low-powered design. This capability enables real-time adjustment of important modulation parameters during operation and allows for the utilization of the same electronics for implementing a variety of different types of modulation when needed later, facilitating the development of acoustic communication systems. For modulation protocol, the Binary Frequency Shift Keying (BFSK) modulation scheme is implemented, as it is a simple, robust, and reliable modulation scheme that can be used in harsh environments [14].

**Figure 4:**
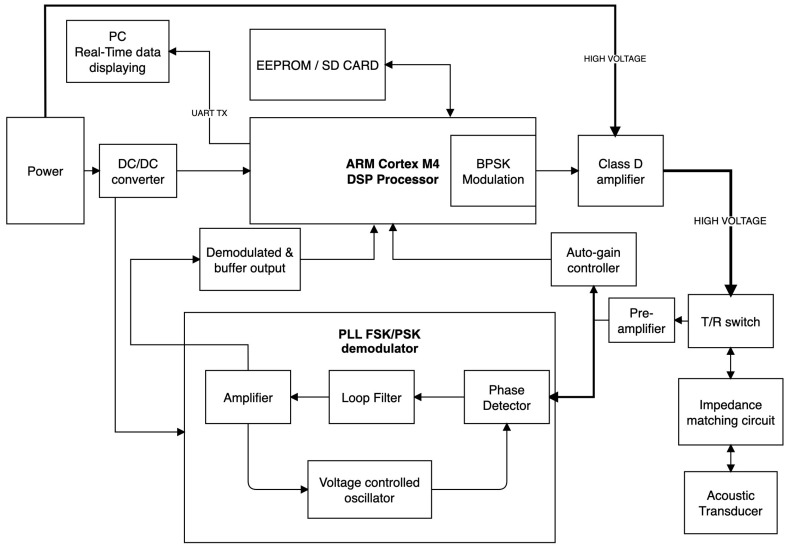
Block diagram of the acoustic communication system.

The block diagram of the acoustic communication system is shown in Figure 4. The transmit channel is composed of the frequency generator, the power amplifier, and the impedance matching circuit. The receive channel is made up of the transducer as a source, protection circuitry (T/R switch), a pre-amplifier, an auto-gain control circuit, and a phase lock loop (PLL) circuit. The operation of acoustic communication is as follows: In transmit mode, the sensor or image data are converted to ASCII 8-bit integers, and a start and stop bit is added to the beginning of the set of integers to track the state of communication between two acoustic data links. Then, the onboard microcontroller converts the logical information (0 and 1) into an analog signal, amplifying and modulating it and sending it to the transmitter. Next, the modulated analog signal is converted into an acoustic pressure wave, and the wave propagates through the acoustic channel. In receive mode, the received analog acoustic signals are pre-amplified, PLL demodulated (carrier synchronization), sampled, and processed onboard in real-time, converting them back into the original digital information through signal processing. The received analog signal is then stored in EEPROM or on an SD card for debugging purposes, and/or the data are output by the UART TX pin for real-time monitoring. The signal recording system is configured with a sampling rate of 50 kHz, allowing for the detection of frequencies up to 25 kHz.

For the onboard microcontroller, a STM32f405 processor is used to program different TX modulation signals as well as high-speed digital signal processing. While BFSK demodulation relies on a dedicated PLL chip, the processor is additionally configured to buffer and record both the pre-amplified analog signal and the PLL-demodulated signal onto an external flash drive for debugging purposes.

## 4. Experimental Test

**Figure 5:**
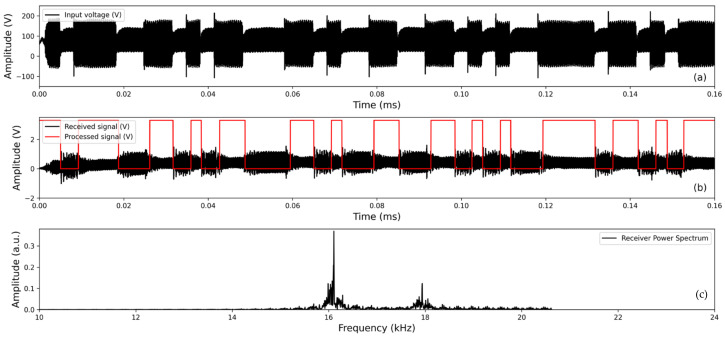
The figure shows the measured transmission input voltage (**a**), received and processed decoding signal (**b**), and power spectrum of the received signal (**c**).

To evaluate the drive electronics and applicability of acoustic transducers for a high-bitrate communication system, experiments involving the transmission of a BFSK signal with various data rates were conducted in laboratory-grown ice with a diameter of 0.54 m and a height of 1.27 m. The data were characterized by generating a train of binary signals and detecting the received signals. In this process, the 0 bit is set at a frequency of 16 kHz, while the 1 bit is designated at 18 kHz. Note that in this test, a higher frequency of 16 kHz was used instead of 13.7 kHz for the representation of 0 bits. This adjustment was made due to the limitations imposed by the size of the laboratory ice testbed and the longer wavelengths associated with lower frequencies. Figure 5 shows an example of the transmitted and received/processed acoustic signals in ice. The power spectrum of the received raw signals is also included, highlighting the transmitting frequencies of 16 kHz and 18 kHz.

**Figure 6:**
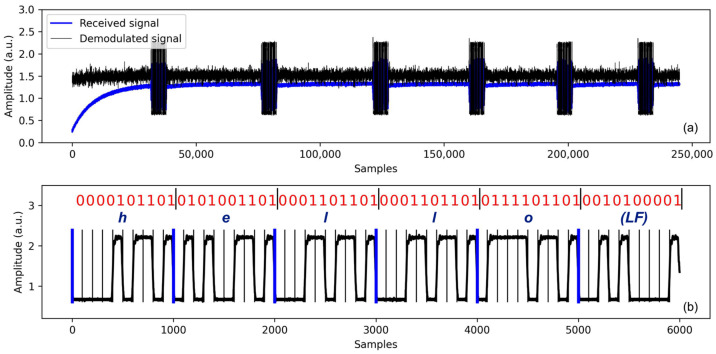
The figure on the top (**a**) shows the receiving and demodulated signals in an ice medium. The device transmits blocks of data at 1-s intervals between characters, and the receiver captures the transmitted data. The figure on the bottom (**b**) is a zoomed-in view of the demodulated signals from the initial packet of the received signal.

Figure 6 shows the recorded acoustic signals during transmission and reception in ice using the developed acoustic communication system. Note that when extended bits (beyond 50) are transmitted through the ice, interferences occur due to the reflections from the surrounding container wall. Therefore, throughout the test, the transmission length of the bit stream (a few bytes) was constrained to avoid interferences caused by ringing and reflections from the surrounding container wall. For this test, the acoustic signal was modulated using the UART protocol at a rate of 300 bits per second (BPS), transmitting the ‘hello’ message represented as [01101000 01100101 01101100 01101100 01101111]. Each byte was transmitted with the inclusion of a start bit (1) and a stop bit (0). Note that, from the figure, the signal should be interpreted in reverse order. The results show that the modulated acoustic wave signal traverses through the ice with minimal dispersion and interference. Transmission and reception of the data at speeds up to 500 BPS were achieved in this test with negligible errors.

**Figure 7:**
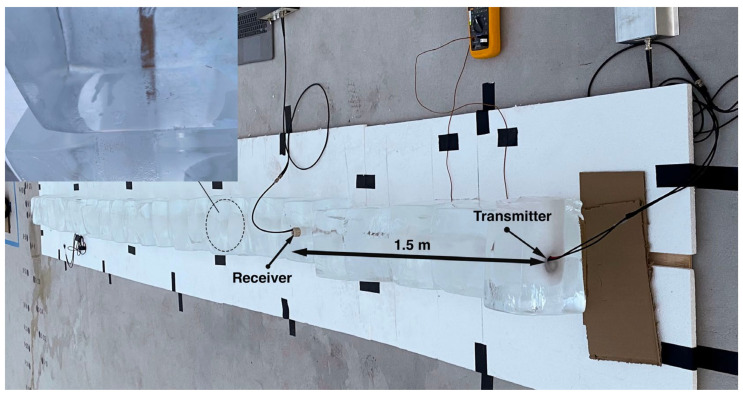
The ice testbed showing the location of the transmitter and receiver. The inset highlights the contact issues encountered, revealing irregular and sporadic points of contact between the ice blocks.

For a long-range ice communication test, the ice testbed was arranged by stacking ice blocks, as shown in Figure 7. Unfortunately, the test did not yield the anticipated results, primarily due to the improper fusion of ice blocks. The photo in Figure 7 shows the ice test setup. Owing to the non-uniform flatness of the ice block surfaces, only a limited number of sections make sporadic contact with one another. Consequently, the measured attenuation is significantly higher than expected, with no signal reception beyond a 1.5-m communication distance. The test results indicate that acoustic attenuation is significantly influenced by the mechanical integrity of the ice medium, and the ice in this test setup is an inadequate analogue for Europa’s ice, highlighting the necessity for a field test that reflects Europa’s deep, high-pressure ice environment.

Tests of the acoustic communication breadboards were conducted in an air environment due to the challenges associated with creating a defect-free ice testbed exceeding 10 m. This approach allowed the functionality of the drive electronics and communication capability to be evaluated in an open-space environment, free from interference. Similar to the lab test shown above, the tests involved the transmission of ASCII data byte image files using a frequency-shift keying (FSK) signal, maintaining an acoustic pressure level of approximately 100 dB re 20 µPa for each data transmission. The results reveal successful signal detection at distances exceeding 55 m in the air, which is determined by sound meter readings indicating signal strengths of around 55.4 dB at 55 m. The results show a successful transmission and reception of various image data with a bit error rate (BER) below 1% at a transmission rate of 500 BPS and 0% BER at rates below 300 BPS over a 2-m distance in the air. The test results indicate that both the acoustic transducers and driving electronics are operating as expected.

## 5. Field Test

**Figure 8:**
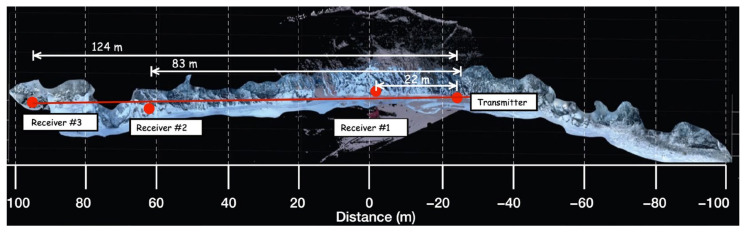
Acoustic test setup on Matanuska Glacier showing the location of the transmitter and receiver.

Following the verification of the functionality of the developed acoustic communication system, acoustic communication tests were performed at the Matanuska Glacier, Anchorage, Alaska. Figure 8 shows an overview of the field site, indicating the positions of acoustic transmitters and receivers with their respective distances. The distance and alignment of the acoustic transmitter and receivers were determined using a Leica BLK360 LiDAR scanner. This scanner offers 360-degree horizontal coverage and 270-degree vertical coverage, with a spatial resolution ranging from 25 to 50 mm at a distance of 10 m. It achieves an accuracy of 4 mm at 10 m. Note that due to the dimensions of the wall exceeding the range of a single scan, multiple scans were conducted and realigned. The optimal nine scans were used for the reconstruction of the glacial wall.

**Figure 9:**
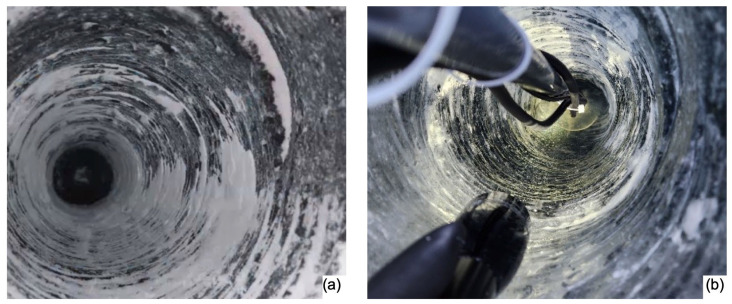
The transducers’ placement on the wall of the Matanuska Glacier, showing a drilled hole (**a**). The image on the right (**b**) shows the acoustic transducers, fully refrozen after being filled with cold water.

To achieve acoustic coupling of the transducers within the Matanuska glacier wall, the ice wall was drilled to create 8-inch-diameter holes that are roughly 1.2 m deep. The holes were drilled at an angle from the horizontal axis of the glacier ice wall. Subsequently, the transducers were placed, aligning their axes parallel to the wall’s surface. The drilled hole was then filled with cold (2~5 °C) water, ensuring complete coverage of the transducers, which were left to freeze overnight. The following day, the transducers were tested to assess their performance. Figure 9 shows photos of the drilled holes and transducers embedded in the ice.

**Figure 10:**
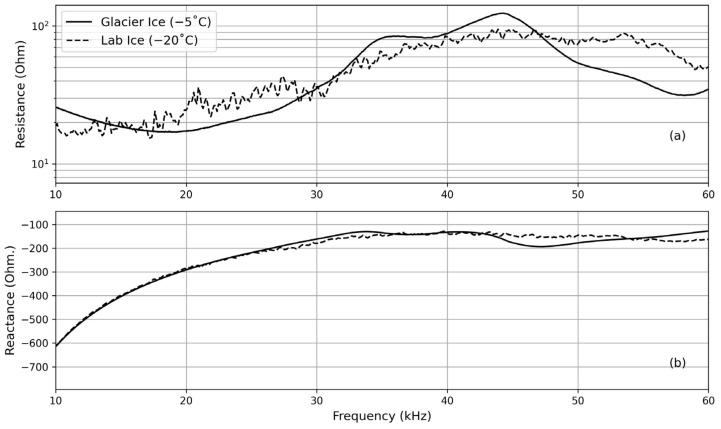
Comparison of the electrical resistance (**a**) and the reactance (**b**) of the acoustic transducers in the glacier and in the lab ice.

Before the acoustic communication test, the electrical characteristics of acoustic transducers were investigated as a function of frequency to make sure there were no distinctions between the glacial ice and the laboratory ice. The electrical responses of acoustic transducers in the field as a function of frequency are shown in Figure 10, in comparison with those measured in the laboratory, confirming that the electrical characteristics of the transducer in glacier ice are similar to those observed in the laboratory.

**Figure 11:**
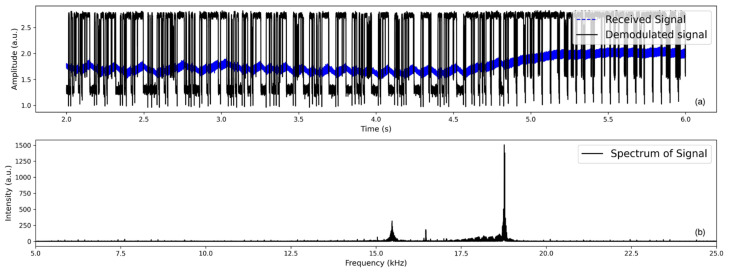
Recorded received and demodulated byte signals in glacier ice at a distance of 22 m from the transmitter (**a**) and the power spectrum of the signal (**b**).

In the initial test, the bit streams were transmitted into the glacier ice, and the received signals were recorded. As expected, the overall characteristics of received acoustic signals in glacial ice were similar to those observed in laboratory testing. At a distance of 22 m between transmitter and receiver #1, there was a little acoustic signal interference and multipath propagation effects. All acoustic signals transmitted through the glacier ice were successfully received, and the byte data and acoustic images were reconstructed with minor code modifications, achieving a bit rate of up to 500 bits. The signals of image data transmission through the glacier ice are shown in Figure 11. This involved converting the image into a binary 40 × 40 pixel byte, which was then transmitted through the ice.

**Figure 12:**
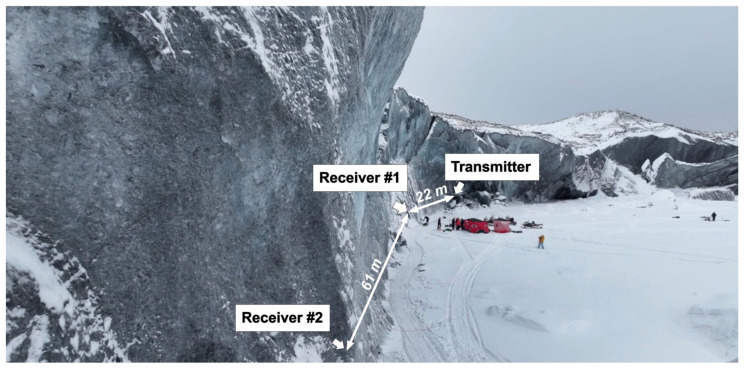
Acoustic test setup on Matanuska Glacier showing the concave curvature of the glacier ice.

**Figure 13:**
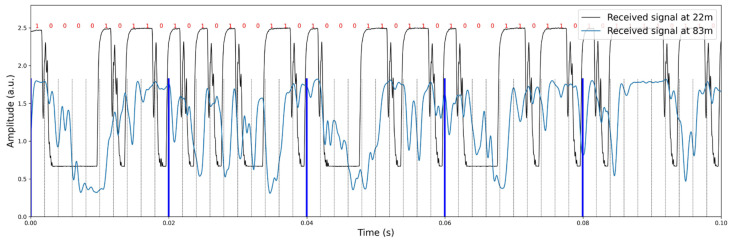
Comparison of the received demodulated signals from the 22 m and 83 m transmission paths. The two experiments were conducted with an identical configuration.

The communication test was also performed at a distance of 83 m between the transmitter and receiver #2. Figure 12 shows a picture of the communication test setup. Unfortunately, the communication path is not straight due to the concave curvature of the glacier ice-exposed surface. A depth of more than 1.2 m was drilled for optimal alignment between the transmitter and receivers; however, uncertainties remain regarding the transducer alignment as well as potential scattering effects from the curved glacier. Despite the concave curvature of the glacier ice, data transmission and reception were achieved at a distance of 83 m. However, at this distance, multipath propagation effects were observed as the transmitted signals traversed multiple paths to reach the receiver, leading to inter-bit interference. Additionally, the received signal at 83 m exhibited a time spread in acoustic wave propagation from source to receiver, resulting in an increased bit-error rate. The comparison of received demodulated signals at 22 m and 83 m is shown in Figure 13.

**Figure 14:**
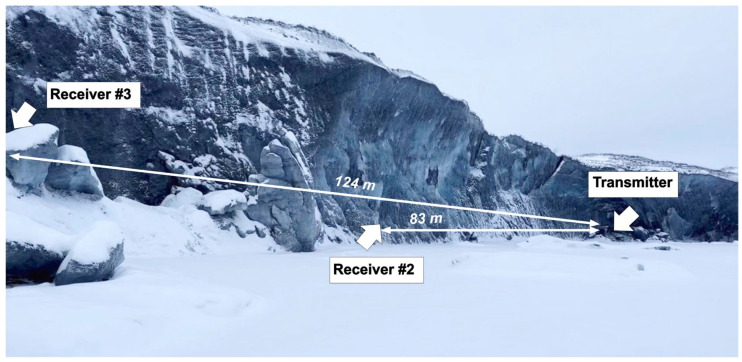
Photo of the acoustic communication test site where the receiver was located on the opposite side of the glacier ice.

At a distance of 124 m (receiver #3) from the transmitter, several missing and damaged packets were encountered, resulting in an inability to reconstruct the data. Only simple pulse/tone-based signals at frequencies of 13~14 kHz were received. The corruption of the acoustic signal during propagation is attributed to scattering and reflection from the curved glacial wall. It is important to note that beyond 83 m (receiver #2), the path requires ascending a hill to gain access to the opposite side of the glacier, as is shown in Figure 14. While this provided a nearly straight communication path, there were several interference objects that could affect communication signals between 83 m and 124 m. After the transducer testing was completed, the ice around the transducers was melted, all transducers were removed from the glacier wall, and the holes were filled with ice. All equipment was retrieved from the test site, and it was left as found before testing.

## 6. Acoustic Attenuation

The attenuation coefficient of Matanuska glacier ice was analyzed by transmitting sine bursts and measuring the received sound amplitudes at various receiver locations according to the equation [15]:

(1) $$\alpha \left(f\right)=\frac{\ln\left(V_{1}d_{1}/V_{2}d_{2}\right)}{d_{2}-d_{1}}$$

where α is the attenuation coefficient with units of dB/m, *V* is the average amplitude value of the received signal at the receiving transducer voltage, and *d* is the distance between the transmitter and the receiver with a unit of m. Subscripts 1 and 2 represent values at distinct receiver locations. Note that this attenuation equation assumes perfect alignment between the transmitter and receiver within a precisely defined distance, as well as ideal coupling between the refrozen transducers and the ice. Unfortunately, there are uncertainties regarding the accuracy of the distance between the transmitter and receivers, the coupling of individual transducers to the ice, and the scattering or reflection effects from the glacier’s curved surface. These factors contribute to the difficulty of obtaining precise attenuation measurements. Matanuska glacier ice has a clear and dense appearance; however, it is relatively warm ice. At a depth of 1 m, the measured temperature of the glacier ice is approximately −5 °C, even though the outside air temperature is around −20 °C. In addition, our observations show that there are air bubbles at various scales, along with some rock inclusions. These factors might contribute to the levels of scattering.

**Figure 15:**
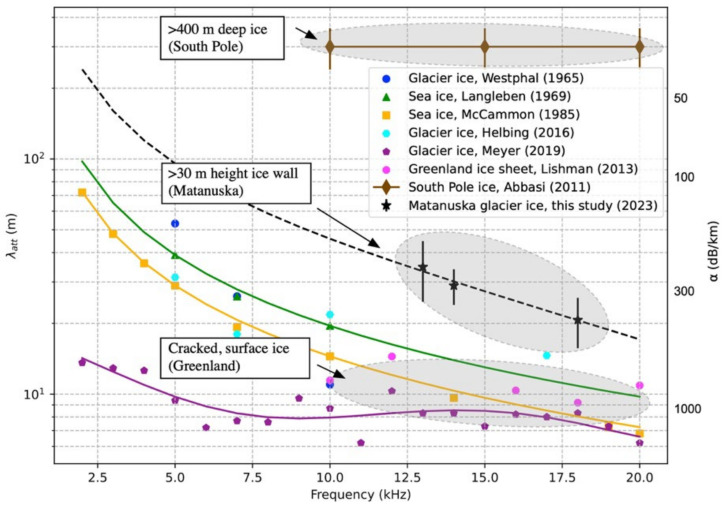
Acoustic attenuation length measured in Matanuska glacier ice in comparison with available data for glacier ice and sea ice. The attenuation length in South Pole ice is ~300 m between 10 and 30 kHz [13,15,16,17,18,19,20].

Figure 15 shows the measured attenuation data in comparison with available attenuation data measured from various ice sources, such as temperate, shallower glacier regions [15,16,17] and sea ice [18,19]. The associated error for each data point represents the range of fluctuations within the measured data. The determined attenuation length of Matanuska glacier ice, derived from the measured signals, is within the range of approximately 25 to 40 m (200 to 400 dB/km) for frequencies between 13 kHz and 18 kHz. These values are higher than the reported attenuation lengths except for South Pole ice data, where most data are in the tens of meters in the frequency range between 10 kHz and 30 kHz, indicating smaller attenuation compared to surface glacier ice and sea ice. Note that the acoustic lengths of South Pole ice are considerably higher than our measurements and other attenuation length data, where the attenuation lengths are reported to be ~300 m with 20% uncertainty in the 10–30 kHz frequency range [13].

The results are not entirely unexpected, considering the mechanical properties of ice are largely influenced by environmental conditions [20,21,22]. Acoustic attenuation data, including our measurements shown in Figure 15, were collected close to the pressure melting point of the ice and the crack near the ice surface, while the South Pole attenuation data were measured at a great depth from the ice surface between 190 m and 500 m, with much colder temperatures of −51 °C to −46 °C and fully annealed porosity. Consequently, the attenuations from temperate shallow glacial ice and sea ice are expected to be higher compared to South Pole ice attenuations due to the higher contents of porosity and air bubbles. Applying a similar methodology [15], the frequency-dependent attenuation coefficients (*A, B*) were derived through a curve fitting method with the measured acoustic signals, resulting in *A* = 0.018 dB/m/kHz and *B* = 9.18 × 10^−7^ dB/m/kHz^4^ with the equation of the form:

*α*(*f*) = *A* *f* + *B* *f*
^4^ (2)

**Figure 16:**
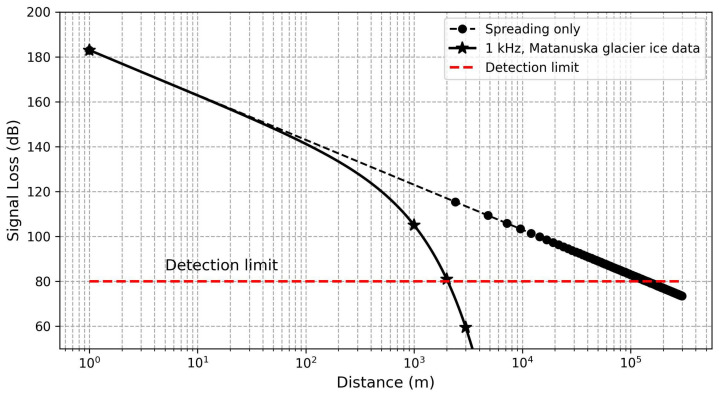
Estimated signal level vs. distance for a 10 W, 1 kHz omnidirectional source.

Figure 16 shows the estimated signal loss from a 10 W, 1 kHz acoustic source against distance, based on the determined frequency-dependent attenuation coefficient, while considering a conservative detection limit of 80 dB due to signal noises at 1 kHz. The figure also includes the signal loss without absorption/scattering effects (i.e., spreading loss alone). The results suggest long-distance (more than 1 km) acoustic communication in ice is potentially possible at frequencies near 1 kHz.

## 7. Conclusions

In this work, the progress in the design and testing of subscale prototypes for acoustic communication through ice is presented. Emphasis has been placed on the feasibility study of utilizing acoustic transceivers for wireless communication through deep ice. The results demonstrate the feasibility of wireless data transmission through ice, marking a significant milestone in ice-based communication technology. Acoustic transducers and drive electronics were designed and fabricated, tailored specifically for operation in ice environments. Through laboratory and field tests, the viability of acoustic signals for ice communication was validated, with successful transmission of data and image files in the 13–18 kHz communication band. The attenuation characteristics of Matanuska glacier ice were also determined. Assuming Europa’s deep ice is relatively pure and lacks cracks and rocks within the range of 10 km to 40 km, much lower acoustic attenuation is anticipated, as is shown in the South Pole ice. Therefore, enabling communication over many kilometers of ice thickness could be feasible by employing reduced transmitting frequencies. However, due to the high variability in published attenuation data and their sensitivity to ice grain structure and porosity, high-priority future work should characterize communication performance and attenuation in a deep refrozen borehole—highly analogous to the expected channel on icy moons. The design and development of low-frequency (less than 1 kHz) acoustic communication devices, along with the formulation of a field test plan for long-distance (more than 10 km) communication, are currently being explored, and the results will be discussed in future publications.

## Data Availability

The data that support the findings of this study are available upon reasonable request.
